# Molecular analysis of genetic diversity in population of *Silybum marianum* (L.) Gaertn in Egypt

**DOI:** 10.1186/s43141-019-0011-6

**Published:** 2019-12-03

**Authors:** Marwa Hamouda

**Affiliations:** Department of Botany, Faculty of Science, Tanta University, Tanta, Egypt

**Keywords:** *Silybum marianum*, Genetic diversity, Protein electrophoresis, RAPD, ISSR

## Abstract

**Background:**

*Silybum marianum L.* Gaertn is a medicinal plant of unique pharmaceutical properties in the treatment of liver disorders and diabetic nephropathy. Biochemical (SDS-PAGE) and molecular markers such as randomly amplified polymorphic DNA (RAPD) and inter-simple sequence repeats (ISSR) technologies were used in this work to detect genetic diversity of 14 collections of *Silybum marianum* population in Egypt.

**Results:**

The electrophoretic pattern of seed protein gave different molecular weight bands, ranging from 24 to 111 KDa with the presence of unique bands. RAPD results revealed a high level of polymorphism (73.2%) using 12 RAPD primers, but only eight of them gave reproducible polymorphic DNA pattern. Sixteen primers were used in the ISSR method; only ten of them yielded clearly identifiable bands. The percentage of polymorphism is about 80% of the studied samples.

**Conclusion:**

The obtained data confirmed that SDS-protein, RAPD, and ISSR markers are important tools for genetic analysis for *Silybum marianum* and recommended to give accurate results.

## Background

Milk thistle *(Silybum marianum (L.)* Gaertn. is an annual or biennial species belonging to the Asteraceae family; it is a common weed found in temperate American countries, Australia, and areas of Mediterranean climate regions [1] In Egypt, it grows wild in most districts especially in the Nile Delta. The fruit’s extracts of this plant, exhibit several medicinal properties [2, 3]. This species is normally cultivated for the production of silymarin [4, 5] which is used for treating liver disorders [6]. Silymarin is also beneficial for reducing the risk of developing certain cancers [7]. The seeds and whole plant biomass can be used for oil and bioenergy production, respectively [1, 8]. The genus *Silybum* comprises of two species: *S. marianum* and *S. eburneum* [9].

Seed storage proteins are highly independent of environmental fluctuations. Sodium dodecyl sulfate-polyacrylamide gel electrophoresis (SDS-PAGE) technique is commonly used for separation of seed storage proteins [10]. The analysis of SDS-PAGE is one of the practical methods to study evolutionary relation of plants [11]. This type of technique has been used to analyze genetic diversity among different species of plants [12, 13]. The seed protein profiles reflect genetic affinities within a taxon and even between different biological entities [14].

The determination of genetic diversity within and among populations is great importance for the improvement of medicinal plants. Furthermore, the identification of genetic relationships among populations or genotypes is essential for the efficient utilization of plant genetic resources [15]. Molecular techniques provide effective tools for comprehensive genetic analysis of diversity and population structure [16]. DNA markers are reliable because the genetic information is unique for each species and is independent of age, physiological conditions, and environmental factors [17]. The information derived from the DNA further provides a great number of characters (markers) that are easy to observe, score, and analyze independent of the growth stage, season, location, and agricultural practice [18].

The use of randomly amplified polymorphic DNA (RAPD) technique for the study of genetic variation has been demonstrated as suitable in many species [19]. RAPD is a relatively recent technique and has been widely used for the estimation of genetic relationships in various crops of agronomic importance due to its low cost; its assay is rapid and easy and needs a small amount of plant material with prior sequence information [20, 21]. Recently, various DNA-based methods have been successfully used for the pharmacognostic characterization of medicinal plants and herbal medicines for the purpose of quality control and standardization [22].

The inter-simple sequence repeats (ISSR) developed by Zietkiewicz et al. [23] to access variation in the numerous microsatellite regions dispersed throughout the various genomes (particularly the nuclear genome) circumvents the challenge of characterizing individual loci that other molecular approaches require. The ISSR has been used with success to identify and determine relationships at the species, population, and cultivar levels in many plant species, including several aromatic and medicinal plants [16, 24–28]. Sharaf et al. [29] studied 12 *S. marianum* accessions and reported that it is not possible to differentiate between the tested populations based on one identification system alone. Therefore, they applied combined class pattern based on protein, isozyme, RAPD, and ISSR to obtain a better resolution. Several reports indicated the importance of using more than one class of molecular marker to assess the genetic diversity of many species. It is evident from these reports that combined molecular investigations are beneficial than any individual analysis system. Thus, the objective of the present study was to evaluate the genetic diversity among 14 samples of *S. marianum* collected from different locations in Egypt using biochemical protein electrophoresis (SDS-PAGE) of seed protein and DNA molecular marker (RAPD-PCR) of total genomic DNA.

## Methods

### Plant material and collection sites

Seeds of wild *S. marianum* plant were collected from different sites in Egypt at a different altitude, during April and May (2013–2014). GPS of the collection sites is shown in Table 1. The identification and nomenclature of the studied plants were carried out by Prof. Kamal Shaltout, Flora and Taxonomy Unit, Faculty of Science, Tanta University. The Voucher specimens have been deposited in the Herbarium of Botany and Microbiology Department, Faculty of Science, Tanta University, with the voucher number “TAN-2014-28”. The genetic variations using RAPD and protein electrophoresis were studied among the collected seeds.

**Table 1:** GPS of the sites of *Silybum marianum* studied samples

Samples no.	Collection sites	Governorate	Latitude (N)	Longitude (E)	Habitat
1	Beni Sewif	Beni Sewif	29° 09′ 04.4″	030° 59′ 07.6″	Canal edge
2	El-Fayuim road	El-Fayuim	29° 23′ 28.0″	030° 52′ 24.1″	Field edge
3	El-Fayuim	El-Fayuim	29° 21′ 37.3″	030° 41′ 05.1″	Road side
4	Kalama	El-Kalubia	30° 13′ 45.2″	031° 12′ 02.9″	Road side
5	El-Monofeia road	El-Monofeia	30° 34′ 17.6″	031° 07′ 41.5″	Road side
6	Tala	El-Monifeia	30° 40′ 08.1″	030° 56′ 55.6″	Field edge
7	Tanta	Gharbia	30° 48′ 29.5″	030° 52′ 35.2″	Field edge
8	Kafr El-Dawar	Gharbia	31° 07′ 36.4″	030° 08′ 35.4″	Road side
9	Saft El-Horeia	El-Behera	30° 56′ 27.1″	030° 35′ 23.5″	Road side
10	Abis	Alexandria	31° 12′ 8.0″	030° 01′ 04.9″	Field edge
11	El-Amereia	Alexandria	31° 01′ 3.3″	029° 47′ 44.2″	Field edge
12	Borg elarab	Alexandria	30° 56′ 58.6″	029° 36′ 24.3″	Road side
13	El-Mansoura	El-Dakahlia	31° 03′ 50.0″	031° 21′ 56.6″	Road side
14	Kafr El-Shiekh	Kafr El-Shiekh	31° 03′ 46.7″	030° 57′ 27.0″	Canal edge

### Protein analysis

For protein extraction, seeds of 14 sample plants were ground to fine powder according to the protocol of Payne and Corfield [30] and proteins were extracted in Tris-HCl buffer-pH 8 containing 5% glycerol and 0.1% β-mercaptoethanol. The extracted protein solutions were resolved in 12.5% polyacrylamide gel using a Pharmacia low molecular weight standard molecular weight marker in a Cole Parmer vertical gel electrophoresis apparatus (Model SE400). At the end of electrophoresis, protein bands were revealed by Comassie Brilliant Blue R-250 staining and destained by methanol and acetic acid solution for overnight. The gel was then photographed with a Kodak digital camera Model AF3X optical aspheric lens, 9.2 megapixel, and molecular weight for protein bands was calculated using the Lab Image software version 2.7 produced by Kapelan GmbH, Germany.

#### DNA extraction

DNA was extracted from leaves of 2-week-old M2 seedlings, grown in pots in the laboratory at 20 °C, using the DNA-easy Plant Mini kit (Qiagen, USA, Cat. # 69104) as described in the instruction manual. The quality and quantity of the extracted DNA were measured using nano-drop 2000C (Thermo Scientific) and its integrity was tested in 1% agarose gel. DNA concentration in all samples was adjusted to 25 ng/μl for PCR reactions.

### RAPD analysis

For RAPD fingerprinting, as outlined by [19], a total of 12 random primers were tested for production of RAPD fragments, only eight of them revealed clear readable and constant profiles (Table 2). The RAPD-PCR procedure was performed as described in Sambrook and Russell [31] in a total of 25 μl reaction mixture as the following 1 μl DNA (25 ng/), l μl MgCl2 (50 mM), 2 μl primer (10 nM), 12.5 μl Bio-Mix Red (GE Healthcare UK Limited, Amersham, UK), and 8.5 μl of nuclease free water. The applied amplification condition was as follows: 95 °C for 5 min as initial denaturation followed by 40 cycles of 95 °C for 30 s., 38 °C for 45 s., and 72 °C for 1 min. The PCR products were left at 72 °C for 15 min for final extension. The PCR products were screened using 1.5% agarose gel.

**Table 2:** The sequence of the oligonucleotide primers used for the RAPD-PCR analysis

Primer code	Nucleotide sequence 5′-3′	Primer code	Nucleotide sequence 5′-3′
L12	GGGCGGTACT	OPG-03	GAGCCCTCCA
L13	ACCGCCTGCT	OPP-01	GTAGCACTCC
L20	TGGTGGACCA	OPP-10	TCCCGCCTAC
O-17	GGCTTATGCC	X06	ACGCCAGAGG
OPC-17	TTCCCCCCAG	Z05	TCCCATGCTG
OPG-01	CTACGGAGGA	Z-18	AGGGTCTGTG

### DNA extraction and purification

For genetic variability analysis, the bulk seeds from each of 14 plant samples were collected and washed by distilled water and genomic DNA was extracted using Gene JET™ Plant Genomic DNA Purification Kit.

### ISSR

The ISSR fingerprinting was performed using a protocol, developed by Sigma (Biometera Uno thermal cycler, Germany), and does not involve DNA extraction. The procedures recommended by the manufacturer have been followed. In brief, a small disc of fresh leaves taken from actively growing seedlings using a 50-mm Harris Uni-core puncher supported by cutting mat. The disc was added directly into 25 μl PCR reaction mix containing 25 mM MgCl2, 1X PCR buffer, 200 μM dNTPs (Applied Biosystems), 1 U of Taq DNA polymerase (Applied Biosystems, Ampli-Taq Gold), 2 pmole of each primer, and the leaf disc. Polymerase chain reaction was made for amplification of ISSR fingerprinting. The PCR amplification was performed using Bio-Rad thermo-cycler according to the following cycle profile: initial denaturation at 95 °C for 10 min, followed by 35 cycles of 30 s at 94 °C, 30 s at 60 °C and 30 s at 72 °C, and 5 min at 72 °C for final product extension. A total of 16 ISSR primers were used and only 10 primers produced a clear readable profile (Table 3) The PCR products of RAPD were separated in 1.5% agarose gel containing 0.5 μg/ml ethidium bromide using a submarine EC370 Minicell and an EC105 power supply (EC Apparatus Corporation, USA). The DNA size was calibrated against Hyper-Ladder I (GE Health care UK Limited, Amersham, UK). Meanwhile, the ISSR PCR products were separated in 1.8% agarosegel. The DNA size was calibrated against 100 bp ladder (Thermo scientific). The Lab image program version 2.7 produced by Kapelan Bio-Imaging GmbH was used for DNA size determination.

**Table 3:** The sequence of primers assayed in ISSR-PCR

Primer code	Nucleotide sequence 5′-3′	Primer code	Nucleotide sequence 5′-3′
HB-09	GTGTGTGTGTGTGG	809	AGAGAGAGAGAGAGAGG
HB-10	GAGAGAGAGAGACC	814	CTCTCTCTCTCTCTCTTG
HB-11	GTGTGTGTGTGTCC	825	ACACACACACACACACT
HB-14	CTCCTCCTCGC	841	GAGAGAGAGAGAGAGAYC
HB-15	GTGGTGGTGGC	844A	CTCTCTCTCTCTCTCTAC
17898A	CACACACACACAAC	844B	CTCTCTCTCTCTCTCTGC
17899A	CACACACACACAAG	876	GATAGATAGACAGACA
UBC	GTGTGTGTGTGTGTGTC	UBC827	ACACACACACACACACG

The presence or absence of protein, RAPD, and ISSR bands was scored as 1 for presence or 0 for absence of markers respectively for estimating genetic variation. Euclidian distance Romesburg [32] was calculated and used for measuring the similarity between the 14 samples using the software program, Community Analysis Package 4.0 (CAP) developed and was used according to Seaby and Henderson [33]. The dendrogram was constructed based on the similarity matrix data using the unweighted pair-group method with arithmetic averages (UPGMA) clustering and Free Tree software [34].

## Results

### Protein electrophoresis

The SDS-PAGE has been used successfully to investigate the relationships within and between species, genera, sections, and tribes. Table 4 illustrates the SDS-PAG electrophoretic banding pattern of seed protein polypeptides for 14 samples of *S. marianum*. In total, 13 protein bands ranging from 24 to 111 kDa were observed; 10 bands are polymorphic with different molecular weight, 2 are monomorphic, and only one unique band. The unique bands are in sample 3 (El-Fayoum) with a molecular weight of 85.7 kDa.

**Table 4:** The molecular weight obtained from protein electrophoresis of the investigated samples of *Silybum marianum*

No. of band	M.W	Sample number														Band type
		1	2	3	4	5	6	7	8	9	10	11	12	13	14	
1	111.9	0	0	1	0	0	0	0	0	0	1	0	0	0	0	P
2	100.1	1	1	1	1	1	1	1	1	1	1	1	1	1	1	M
3	85.7	0	0	1	0	0	0	0	0	0	0	0	0	0	0	U
4	79.6	1	1	1	1	1	1	1	1	1	1	1	1	1	1	M
5	69.8	0	1	1	0	0	0	0	0	0	1	1	0	1	1	P
6	61.3	1	1	1	0	0	0	1	1	1	1	1	1	1	1	P
7	59.4	0	0	0	0	1	0	1	1	1	0	1	0	1	1	P
8	57.8	0	0	0	0	0	0	0	0	0	0	0	1	1	1	P
9	51.1	1	1	1	1	1	1	1	1	1	1	1	0	0	0	P
10	47.1	0	1	1	0	0	1	1	1	1	1	1	1	1	1	P
11	31.8	1	1	1	0	0	1	1	1	1	0	0	0	0	0	P
12	30.3	0	0	0	1	1	0	0	0	0	0	0	0	1	1	P
13	24.4	1	1	1	0	0	0	0	0	0	1	0	0	0	0	P

*P* polymorphic, *M* monomorphic, *U* unique

### RAPD analysis

**Fig. 1:**
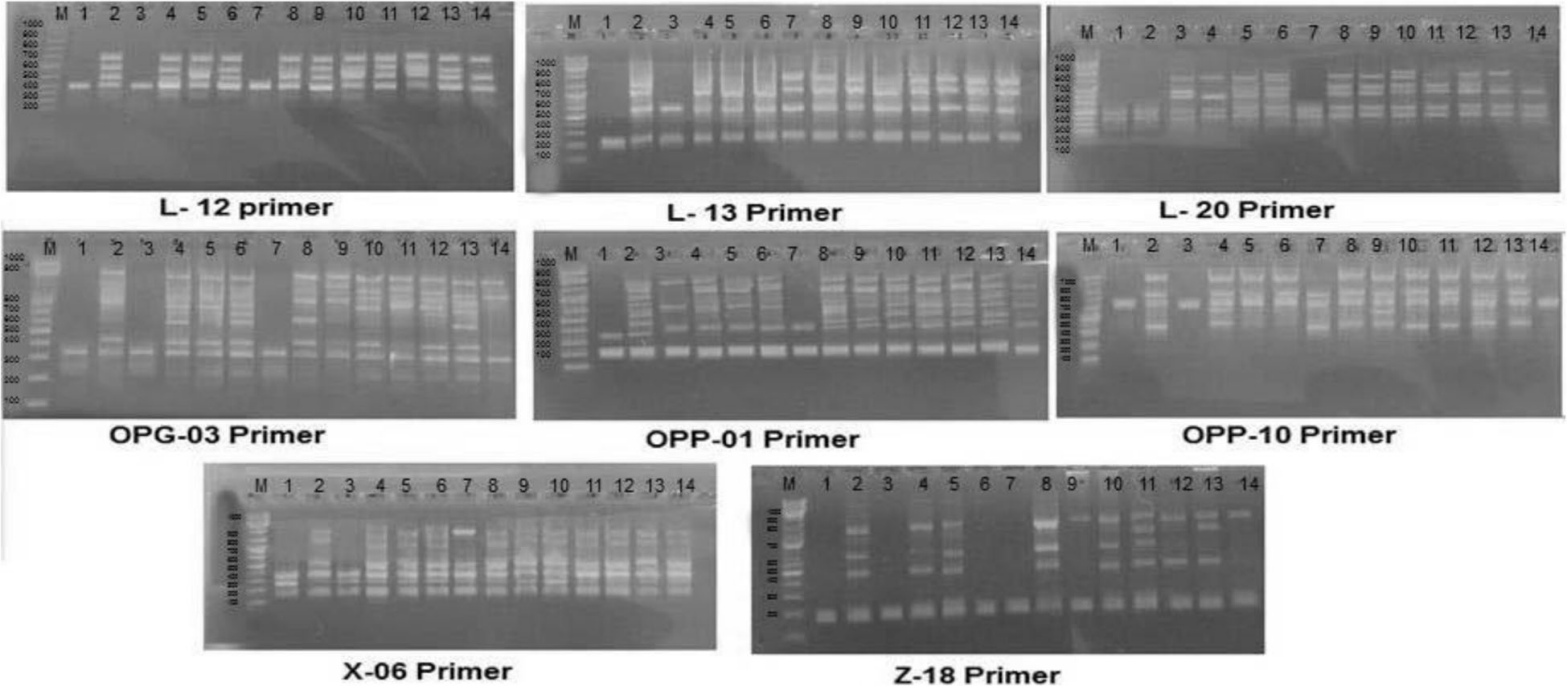
RAPD profile of the 14 samples of *Silybum marianum* generated by eight primer. M: 1 kb Plus DNA ladder

RAPD technique has been increasingly employed for population studies and it provides valuable data on diversity through their ability to detect variations at the DNA level. In the present study, RAPD products were generated from bulked samples separately collected from different sites across Egypt governorates. Figure 1 show the results of RAPD analysis for the 14 *S. marianum* samples by the selected primers. A total of 47 amplified fragments were recorded, of which 34 were polymorphic. The fragment size generated by the tested primers ranged from 196 to 1319 bp. The percentage of polymorphism produced by the eight selected primers was 73.2%. Primer OPP-01 generated the highest number of amplified fragments with the lowest percentage of polymorphism, while the highest percentage of polymorphism (83%) was recorded by OPG-03 (Table 5). The number of amplification products generated by primers varied from 4 to 8 bands and it was primer dependent.

**Table 5:** Total bands, number of monomorphic and polymorphic bands, and the percentage of polymorphism of selected eight RAPD primers among the 14 studied samples of *Silybum marianum*

Primer code	Fragment size (bp)	Total bands	No. of monomorphic bands	No. of polymorphic bands	% of polymorphism
L12	382-690	4	1	3	75
L13	274-888	4	1	3	75
L20	371-880	6	2	4	66
OPG-03	210-880	6	1	5	83
OPP-01	238-944	8	3	5	62
OPP-10	417-1016	5	1	4	80
X-06	196-921	7	2	5	72
Z-18	248-1319	7	2	5	72
Total	-	47	13	34	73.2

#### ISSR analysis

**Fig. 2:**
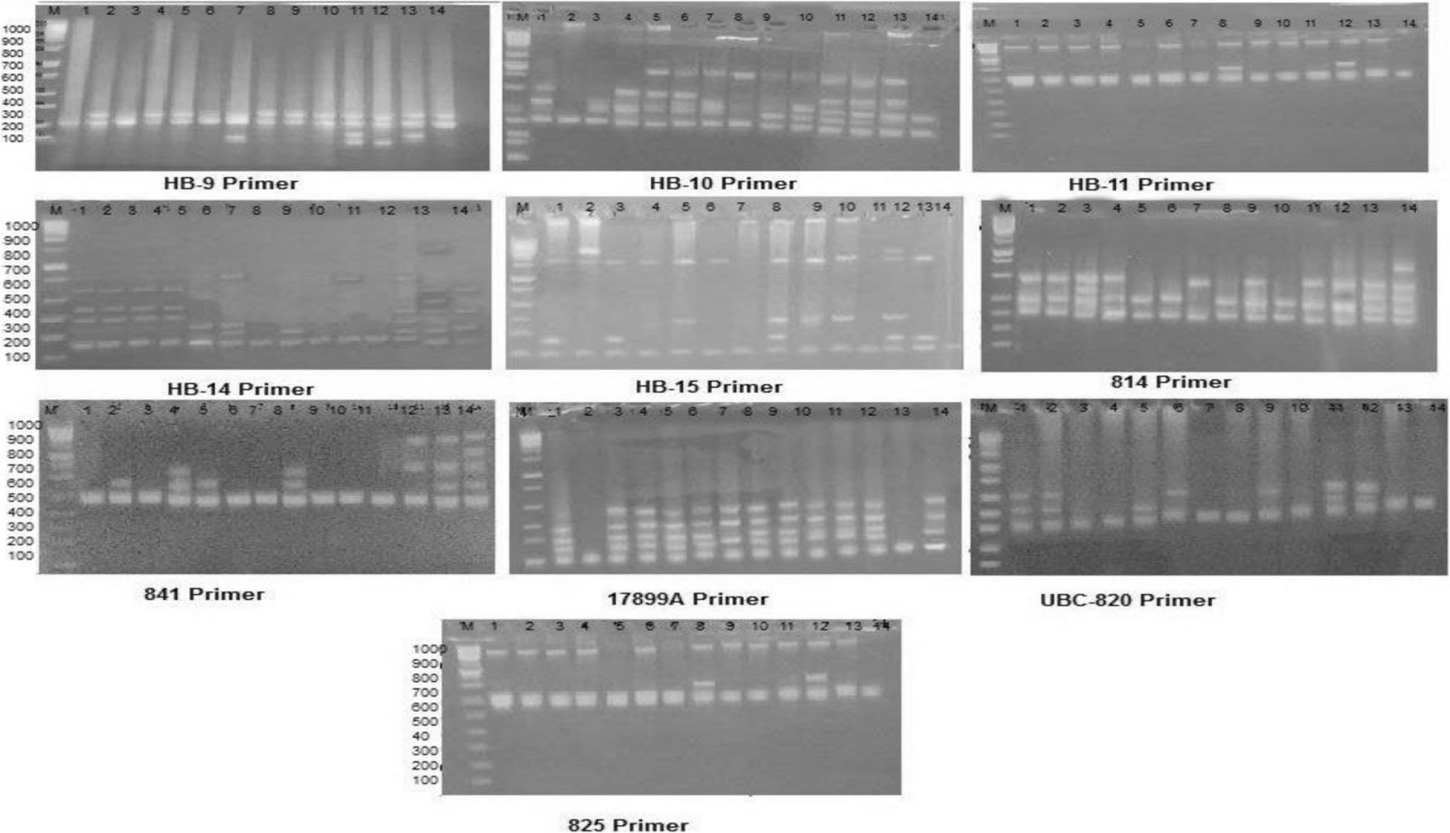
ISSR profile of the 14 samples of *Silybum marianum* generated by ten primers

Out of the 16 ISSR primers used in this study, ten gave rise to reproducible markers. The fragment size generated by the tested primers ranged from 77 to 1498 bp (Fig. 2). Forty-five out of total of 56 markers were polymorphic (79.3%). Primer UBC-820 produced the highest percentage of polymorphism 100% (Table 6), while the smallest polymorphism percent revealed by the primer 814. The number of amplification products generated by primers varied from 4 to 8 bands. The highest number of ISSR markers was 7 and was revealed by HB14 bands.

**Table 6:** Total bands, number of monomorphic and polymorphic bands, and the percentage of polymorphism of the 14 studied samples of *Silybum marianum* generated by ten ISSR primers

Primer code	band size (bp)	Total bands	No. of monomorphic bands	No. of polymorphic bands	Polymorphism (%)
HB-09	77-236	4	1	3	75.0
HB-10	125-987	6	1	5	83.3
HB-11	623-1112	4	1	3	75.0
HB-14	175-600	8	1	7	87.5
HB-15	123-1498	7	1	6	85.7
814	242-555	5	2	3	60.0
825	582-1009	5	1	4	80.0
841	459-1093	6	2	4	66.6
17899A	147-311	5	1	4	80.0
UBC-820	204-1474	6	0	6	100.0
Total	-	56	11	45	79.3

### Combined biochemical and molecular marker

**Fig. 3:**
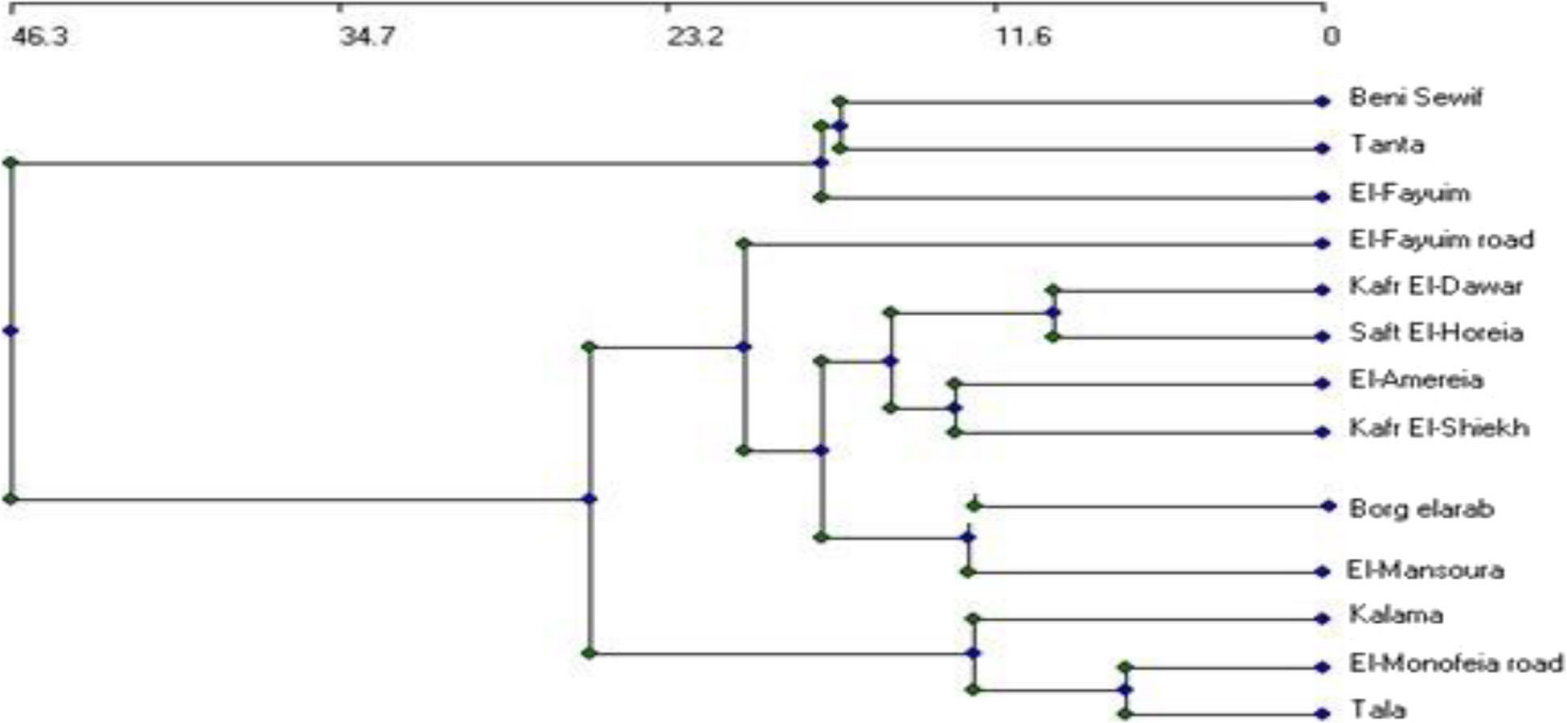
UPGMA dendrogram based on data generated from biochemical and molecular markers, showing the genetic linkage distance among the studied samples of *Silybum marianum*

Combined data of both markers (RAPD, ISSR, and protein) generated a dendrogram that separated the samples into two distinct clusters (Fig. 3). The first one comprises three samples from Bani Sweif, Tanta, and El-Fayoum locations, while the second contains the last samples which were linked together with similarity coefficient 26.4. It is obvious through the dendrogram and Table 7 that the samples of Monofeia road and Tala are closely related with similarity of 81.33%, and the samples of Borg alarab and El-mansoura are closely related with similarity 80.5%.

**Table 7:** Euclidean distance based on biochemical and molecular markers showing the variability among the studied samples of *Silybum marianum*

	Beni Sewif	El-Fayuim road	El-Fayuim	Kalama	El-Monofeia road	Tala	Tanta	Kafr El-Dawar	Saft El-Horeia	El-Amereia	Borg elarab	El-Mansoura	Kafr El-Shiekh
Beni Sewif	1												
El-Fayuim road	0.4651	1											
El-Fayuim	0.5333	0.5155	1										
Kalama	0.3929	0.6022	0.5165	1									
El-Monofeia road	0.3976	0.5579	0.5055	0.76	1								
Tala	0.3902	0.5368	0.5169	0.691	0.8133	1							
Tanta	0.5211	0.5217	0.5732	0.472	0.5663	0.6203	1						
Kafr El-Dawar	0.4	0.6	0.4457	0.688	0.6962	0.6709	0.5556	1					
Saft El-Horeia	0.4096	0.6022	0.5165	0.687	0.6951	0.7125	0.5976	0.7532	1				
El-Amereia	0.4222	0.6495	0.5361	0.678	0.6854	0.6818	0.6136	0.7176	0.72	1			
Borg elarab	0.439	0.6854	0.4787	0.64	0.6867	0.6627	0.6098	0.7215	0.74	0.7273	1		
El-Mansoura	0.4375	0.6517	0.4945	0.663	0.6707	0.6875	0.5926	0.7051	0.7	0.6742	0.805	1	
Kafr El-Shiekh	0.3793	0.6522	0.41	0.607	0.6322	0.6279	0.5227	0.6627	0.66	0.7111	0.714	0.7195	1

## Discussion

The present study deals with establishing a phylogenetic relationship between various samples of the medicinal plant *S. marianum* by using biochemical and molecular marker. In this study, 14 samples of *S. marianum* were collected from different locations from Egypt. Obvious variations were observed between the two constructed dendrograms. To explore the genetic relationship among the studied samples collected from wild populations of *S. marianum,* cluster dendrogram was constructed from combined data of both biochemical and molecular markers. Since when data of the used markers have been linked together and cluster diagram was constructed, the dendrogram separated the studied samples into two distinct clusters. The first cluster consisted of samples from Bani Sweif, El-Fayoum, and Tanta locations. The second cluster comprised the last samples, which were linked together. It was obvious that the samples from Tala and El-Monofeia were more closely related. Several authors have stressed the beneficial application of more than one marker to assess the degree of diversity and relatedness of samples, especially those growing in different sites [35, 36]. Sharma et al. [37] accessed aimed to assess genetic divergence among 16 accessions of *Stevia rebaudiana* Bertoni and to evaluate the comparative efficiency of RAPD and ISSR markers for assessing genetic diversity. The analysis of the combined data set of both techniques clustered the genotypes, based on their geographic locations. However, Idrees and Irshad [38] reported that genetic markers show polymorphism that may be due to a mutation in the genome loci or alteration of nucleotide and make it possible to identify genetic diversity between individual organisms or species. The authors concluded that the high polymorphism obtained indicates that both techniques are efficient for evaluating genetic diversity in the studied samples. The importance of using biochemical markers as seed storage protein and DNA-based marker was reported in numerous studies. The molecular markers are not influenced by the external environmental factors, unlike that of the morphological markers, and hence accurately detect the genetic relationship between the plant species [39]. The advantage of using both biochemical and molecular markers depends on the high stability of seed storage protein that makes it a powerful tool to differentiate and evaluate the origin and the evolutions of cultivated plants [40]. On the other hand, DNA is a source for genetic information and offers great potential for detecting variations on genetic materials level. RAPDs are presumed to result from noncoding regions of DNA [19]. The regions of DNA samples by RAPD technique are expected to be less responsive to selection and to have a higher tolerance to mutations [41]. concluded that both SDS-protein and RAPD markers are equally important for genetic analysis and to evaluate the amount of genetic diversity between the different studied varieties of *Lycopersicon esculentum* L. Also, Osman et al. [42] determined the genetic relationship between some species of *Zea mays* and *Sorghum* using SDS-PAGE of seed protein and RAPD-PCR markers. Phenotypic variation is associated with genetic diversity and dependent on environmental conditions, as well as, on the interaction between genotypes and environment [43]. However, molecular markers have advantages that they provide fast results and detailed genetic differences without interferences from environmental factors.

Thus, it can be concluded that both markers applied in the present study have revealed high polymorphism and genetic diversity among the *S. marianum* plants, which varies between samples from different locations. This may contribute to the differences in the agro-climatic conditions of the sites of samples collection and the type of propagation of the plant, which might have changed the genetic make-up of its populations [44]. This study may translate great knowledge about the relationship among the studied samples as they assess the polymorphism at both protein and DNA levels. It can be concluded from the obtained data and the related previous studies that using more than one marker is an important tool for investigating the genetic diversity and to differentiate between the studied samples. For recent molecular makers, one may gain insight into DNA sequences other than expression products of nuclear coding loci for population genetic structure [45]. ISSR-PCR represents one of the advantageous alternatives to assess genetic diversity. Using molecular markers as ISSR is a powerful tool in the genetic identification and evaluation of the degree of polymorphism among samples within the wild and cultivated plants. In addition to the simplicity, fast, cost-effective, highly discriminative, and reliability [46]. Many ISSR studies of natural populations have demonstrated the hyper variable nature of these markers and their potential use for population-level studies [22, 47]. ISSR targets simple sequence repeats that are abundant and dispersed throughout the genome; thus, it often reveals a much larger number of polymorphic fragments per primer and enabling higher-stringency amplifications, due to the longer ISSR-based primers [22].

In the present study, genetic diversity was investigated among the 14 studied samples of *S. marianum* from different environmental conditions of Egypt. Seven samples were collected from roadside populations, five from field edges, and the remaining two studied samples were from canal edges. Absence of correlation between genetic and geographic distances of populations has been also noted by [48–50]. Vyšniauskienė et al. [50] aimed to investigate the genetic diversity of *Lupinus polyphyllus* populations of forests and abandoned fields using RAPD method. A total of 192 plants were analyzed and the distance between sampled plants in each population was approximately 20–25 m. Neither UPGMA cluster analysis nor principal coordinate analysis revealed population grouping regarding the geographic differentiation between them. The authors suggested that the studied *L. polyphyllus* populations may be characterized by adaptation to local conditions. The authors came to the conclusion that local adaptation leads to the survival of individuals with certain genetic characteristics that ensure the best adaptation to current conditions. Finding of the present study is in accordance with that obtained by Shafie et al. [51], who evaluated the genetic variation between five populations of *Artemisia capillaris* from a different area in Negeri, Sembilan, and Malaysia using RAPD and ISSR markers. They came to the conclusion that the existence of some level of differentiation among the studied populations might be due to different environmental effects including geographical, hydrographical connection, soil, climate, and biotic factors from different districts.

The relationships between the plant performance and genetic variation, and population size and habitat were investigated by several authors for several species. For instance, Vergeer et al. [52] studied the performance of 17 Dutch populations of the perennial *Succisa pratensis* in relation to the population size, genetic variation, and habitat quality. They used the path-analytical model to analyze the possible relationship between those variable and performance. DNA-based markers for the authentication and identification of medicinal plant importance of DNA fingerprinting for the medicinal herbs [53]. DNA fingerprints led to the identification of closely related plant species. DNA is most stable and does not vary seasonally and with the age of plant species [54]. Recently, RAPD and ISSR have been used for the estimation of genetic diversity in different endangered medicinal plant species [53]. Their study showed that both habitat quality and genetic investigation are important for population persistence.

## Conclusion

It was concluded that seed storage protein electrophoresis in combination with molecular DNA markers, RAPD and ISSR, succeeded to investigate the genetic diversity among the 14 *S. marianum* samples, to image fingerprinting for the studied plant, and to highlight the genomic fragments that are site-specific ones.

## Data Availability

The datasets used and/or analyzed during the current study available from the corresponding author on reasonable request.
